# Expansion of a novel endogenous retrovirus throughout the pericentromeres of modern humans

**DOI:** 10.1186/s13059-015-0641-1

**Published:** 2015-04-12

**Authors:** Joseph Zahn, Mark H Kaplan, Sabrina Fischer, Manhong Dai, Fan Meng, Anjan Kumar Saha, Patrick Cervantes, Susana M Chan, Derek Dube, Gilbert S Omenn, David M Markovitz, Rafael Contreras-Galindo

**Affiliations:** Department of Internal Medicine, Division of Infectious Diseases and Programs in Immunology, Cancer Biology, and Cellular and Molecular Biology, University of Michigan, Ann Arbor, MI 48109 USA; Molecular and Behavioral Neuroscience Institute, University of Michigan, Ann Arbor, MI 48109 USA; Department of Psychiatry, University of Michigan, Ann Arbor, MI 48109 USA; Departments of Computational Medicine and Bioinformatics, Internal Medicine, and Human Genetics, and School of Public Health, University of Michigan, Ann Arbor, MI 48109 USA; Department of Internal Medicine, Division of Infectious Diseases, University of Michigan, Ann Arbor, MI 48109-5640 USA

## Abstract

**Background:**

Approximately 8% of the human genome consists of sequences of retroviral origin, a result of ancestral infections of the germ line over millions of years of evolution. The most recent of these infections is attributed to members of the human endogenous retrovirus type-K (HERV-K) (HML-2) family. We recently reported that a previously undetected, large group of HERV-K (HML-2) proviruses, which are descendants of the ancestral K111 infection, are spread throughout human centromeres.

**Results:**

Studying the genomes of certain cell lines and the DNA of healthy individuals that seemingly lack K111, we discover new HERV-K (HML-2) members hidden in pericentromeres of several human chromosomes. All are related through a common ancestor, termed K222, which is a virus that infected the germ line approximately 25 million years ago. K222 exists as a single copy in the genomes of baboons and high order primates, but not New World monkeys, suggesting that progenitor K222 infected the primate germ line after the split between New and Old World monkeys. K222 exists in modern humans at multiple loci spread across the pericentromeres of nine chromosomes, indicating it was amplified during the evolution of modern humans.

**Conclusions:**

Copying of K222 may have occurred through recombination of the pericentromeres of different chromosomes during human evolution. Evidence of recombination between K111 and K222 suggests that these retroviral sequences have been templates for frequent cross-over events during the process of centromere recombination in humans.

## Background

Upon completion of the human genome project, 8% of the genome was found to be composed of human endogenous retroviruses (HERVs). These ancient viruses infected the germline of the primate lineage multiple times over millions of years, eventually entering the human lineage. Relics of these infections remained in the genome and were subsequently transmitted vertically over the generations [1-3]. Other retroviruses entered the human genome after modern humans had split off from other primates, the most recent of which are the retroviruses of the HERV-K (HML-2) family [4,5]. After infection, HERV-K (HML-2) integrated into the germline DNA to form proviruses consisting of four retroviral genes (*gag*, *pro*, *pol*, and *env*), flanked by two long terminal repeats (LTRs). Evolution introduced mutations such as stop codons or deletions rendering these viruses generally defective; approximately 3,000 proviral remnants remain in the modern human genome [6,7]. About 2,500 of these HERV-K (HML-2) elements exist as solitary LTRs (Solo LTR), which originated by recombination between the 5′ and 3′ LTRs of full-length proviruses, thus removing internal viral genes [8]. About 100 of these elements exist in several chromosome arms as full-length HERV-K (HML-2) proviruses [3,9], while several hundred more were found distributed in 15 centromeric regions [10]. Upon integration, HERV-K (HML-2) produces 5 to 6 bp target site duplication sequences on either side of the provirus, some of which were removed through homologous recombination between different HERV-K (HML-2). This process created hybrid proviruses with different flanking target site sequences [11].

HERV-K (HML-2) represents the most recent HERV entrants into the human genome. Some HERV-K (HML-2) proviruses are present only in humans, while being absent in more ancient primates [4], suggesting their entrance into the hominid genome following the divergence of chimpanzees and humans. Indeed, 11 of those HERV-K (HML-2) proviruses are polymorphically inserted among humans [3,9,12]. As the youngest of the endogenous retroviruses, it is not surprising to find that HERV-K (HML-2) is the most transcriptionally active of the endogenous retroviruses [13-19], and under certain circumstances expression of HERV-K (HML-2) RNA, viral proteins, and virus-like particles (VLPs) can be seen, especially in breast cancer, melanoma, and teratocarcinoma cell lines, although these particles have appeared to be incapable of infection [15,20-23]. We have extended these findings by discovering expression of HERV-K (HML-2) RNA, cDNA, proteins, and VLPs in the blood of patients with HIV-1 infection, lymphoma, and breast cancer [9,24-28].

Sequencing of the RNA found in the HERV-K (HML-2) VLPs of patients with HIV-1 infection allowed us to identify RNA transcripts produced from proviruses previously not assigned in the Human Genome Project [9]. We reported that besides the approximately 100 full-length HERV-K (HML-2) found in the human genome [3,9], hundreds more HERV-K (HML-2) proviruses can be found dispersed throughout the centromeres of at least 15 human chromosomes [10]. These proviruses are related to a common ancestor, termed K111, a virus that inserted in the germline of the primate lineage before the split of humans and chimpanzees about 6 million years ago. K111 spread at a slow pace during the evolution of hominins (Neanderthals and Denisovans), but at a higher rate during the evolution of modern humans. We have postulated a mechanism of expansion of K111 proviruses both within and among several human chromosomes by recombination between centromeres during the evolution of hominids. K111 integrated into centromeric repeat CER:D22Z3 elements found in the centromeres of several human chromosomes [10]. CER:D22Z3 elements, along with the proviruses themselves, would have served as templates for recombination and subsequent expansion of K111 among human chromosomes.

In the present study, we report the discovery of another lineage of endogenous retroviruses, termed K222, which can be found spread throughout the pericentromeres of nine human chromosomes, chromosomes that also contain K111 sequences. Evidence suggests that K222 inserted into the germline of the primate lineage about 25 million years ago, after the divergence of New and Old World monkeys. K222 exists as a single copy in the baboon, orangutan, gorilla, and chimpanzee genomes. In contrast, multiple copies of K222 are found in the modern human, but not in extinct hominins, suggesting that the expansion of K222 took place during human evolution through a mechanism of pericentromere recombination. Strikingly, sequence evidence indicates recombination between K222 and K111, further suggesting that recombination/gene conversion took place within centromeric domains, the core and the pericentromere, during human evolution. The presence of the flanking sequence of K222 at the 5′ end and that of K111 at the 3′ end in the recombinant K222/K111 indicates true cross-over events, offering one of the first demonstrations of recombination within centromeres. We have thus elucidated a novel lineage of pericentromeric proviruses hidden in the human genome that may provide insights into pericentromere biology.

## Results

### Discovery of K222 proviruses

**Figure 1:**
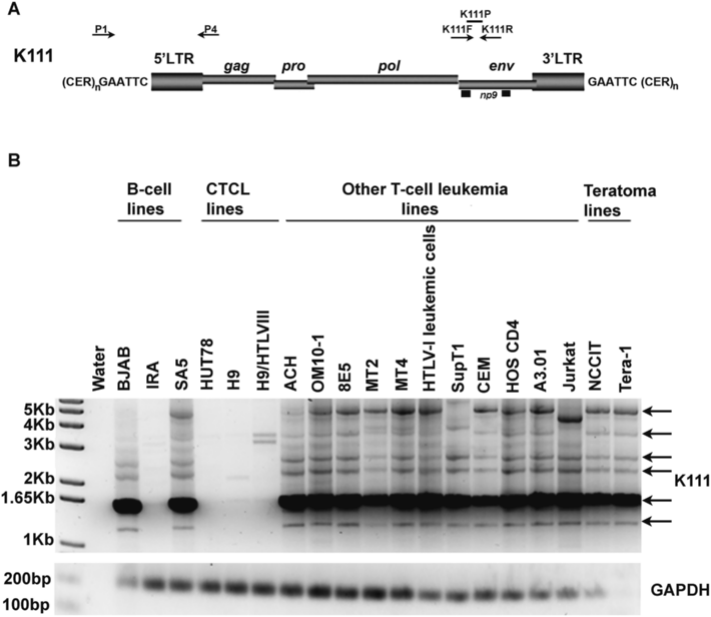
Absence of K111 5′ end in the genome of some cell lines. **(A)** Genomic structure of the K111 provirus. Arrows indicate the position of the primers P1 and P4, which amplify the 5′ integration of K111, and the primer/probe combination K111F, K111R, and K111P that specifically discriminates the K111 and K222 *env* gene from other HERV-K (HML-2) *env* sequences due to a 6 bp mutation [10]. **(B)** Detection of K111 5′ end insertions in human cell lines. The 5′ flanking K111 insertions were detected in all human cell lines tested in this study by PCR using the primers P1 and P4 [10], except for the DNA of cell lines H9, HUT78, H9/HTLVIII, and the IRA B-cell line. Arrows indicate individual K111 insertional polymorphisms. Integrity of the DNA was assessed by amplification of GAPDH (see lower gel). The molecular size of the DNA ladder is shown on the left of the gel. On top of each lane is the name of each cell line subjected to study. The weak bands observed in H9 and H9/HTLVIII were shown by sequencing to be the result of non-specific PCR amplification.

In the present study, we report the discovery of a new lineage of pericentromeric endogenous retroviruses that is found in several human chromosomes. These novel pericentromeric sequences were first identified through the study of three cutaneous T-cell lymphoma (CTCL) cell lines derived from one patient and one B-cell lymphoma line. They were then confirmed to exist in the genome of healthy humans. These cell lines at first appeared to lack the known centromeric endogenous retrovirus K111, which was surprising, as centromeric K111 proviruses are detected by PCR in the DNA of almost all human subjects who have been tested in our laboratory [10]. When we screened for integration of K111 proviruses in the DNA of 19 human cell lines using primers that bind the 5′ flanking sequence in a CER:D22Z3 element and the *gag* proviral sequence of K111 (10; Figure 1A; primers P1 and P4), we detected K111 in all cell lines, but none in one B-cell line (IRA) or in three CTCL cell lines (HUT78, H9, and H9/HTLVIII; Figure 1B). We considered the possibility that the absence of K111 detection was caused by the deterioration of DNA and so we checked for genomic integrity by amplifying another gene, GAPDH. Detection of GAPDH was seen in the DNA of all cell lines tested, suggesting the true absence of K111, or at least the 5′ end of K111, in some cell lines (Figure 1B). Next we screened for K111 by real-time PCR using a set of primers and a custom probe that specifically targets the K111 *env* gene, but no other HERV-K (HML-2) proviruses known at the time (Figure 1A; 10). (We later found this probe detected K222 provirus as well; see below). K111 *env* amplification signal was detected in the DNA of all cell lines tested (data not shown). Taken together, these results indicate that in the genome of some human cell lines, though we were not able to detect the K111 5′ end, we still detect K111 *env*. Lack of detection of the 5′ end of K111 could be explained by deletion of the 5′ portion of the K111 genome in some cell lines and/or deletion/mutation of the sequence that primers P1 and/or P4 target. The persistent detection of K111 *env* signal could otherwise be explained by the presence of an unknown HERV-K (HML-2) sequence, closely related to K111, which could be detected with this primer/probe combination.

To test the above possibilities, we designed a PCR strategy to examine whether incomplete K111, truncated at the 5′ end, or a novel provirus closely related to K111, exists in the genome. Initially, we designed four forward primers that bind the 5′ sequence flanking K111. These primers, in combination with the reverse primer (P4), which binds to K111 *gag*, did not detect K111 5′ end in the DNA of two CTCL cell lines derived from the same individual; all these primers detected the K111 5′ end in most of the normal human DNA (data not shown). This result again suggests the deletion of K111, or at least the 5′ end, in some human cell lines.

**Figure 2:**
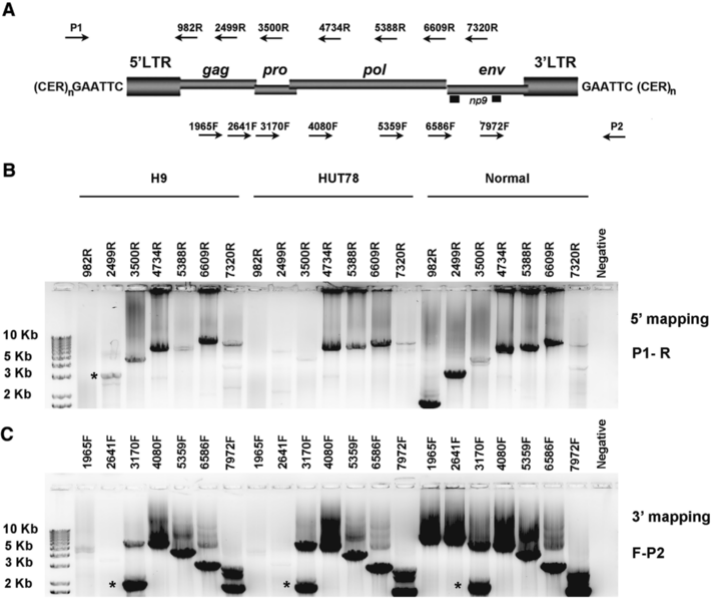
Mapping of K222 proviruses in the human genome. **(A)** Schematic representation of the primer sets used to isolate K222 by PCR. The genomic structure of a centromeric provirus K111 is shown; the viral genes *gag*, *pro*, *pol*, *env*, and *np9*, surrounded by LTRs, integrated into centromeric repeats (CER:D22Z3). The target site duplication of K111 GAATTC is indicated. The primers P1 and P2 bind CER:D22Z3. These primers were used in combination with primers that span the provirus genome. Arrows indicate the position and orientation of the primers; the number above indicates the nucleotide position they bind in reference to K111. Mapping to the 5′ end of the provirus was performed using the primer P1 and a set of HERV-K (HML-2) reverse primers. Mapping to the 3′ end of the provirus was performed with the reverse primer P2 and a set of HERV-K (HML-2) forward primers. **(B, C)** Isolation of K222 provirus. The sequence of K222 was detected by PCR from DNA of the cell lines H9 and HUT78, which lack K111 5′ end. Normal human DNA, containing K111, was used as a control for the PCR reaction. The number shown for each lane represents the primers. The gels show the amplification products of the 5′ mapping **(B)** or 3′ mapping **(C)** of centromeric proviruses in H9, HUT78, and normal human DNA using different combinations of primers. A molecular size ladder is indicated at the left. No amplification products were detected in H9 and HUT78 cell lines, in contrast to normal human DNA, when using the primer sets P1-982R, P1-2499R **(B)**, or primer sets P2-1965F, and P2-2641F **(C)**. An asterisk indicates a band that was shown by sequencing to be the result of non-specific amplification. Sequencing of the mapping products obtained from DNA of H9 and HUT78 cells reveals the sequence of K222.

We next designed a PCR strategy to amplify the centromeric provirus that might exist in the DNA of cells lacking the K111 5′ end. We used primers (forward and reverse) that bind several sites spanning a HERV-K (HML-2) genome in combination with primers (P1 and P2) that bind centromeric regions (Figure 2) [10]. In cells lacking the 5′ end of K111, these sets of primers were able to amplify the genome of a novel provirus, which we term K222. In most normal human DNA, these primers amplify K111 (Figure 2). K222 amplification products were seen only when the complementary primers sit on HERV-K (HMl-2) *pro*, *pol*, and *env* but not the *gag* gene (Figure 2). Cloning and sequencing of full-length K222 revealed two distinct features making K222 different from K111. First, in contrast to K111, K222 lacks the 5′LTR and the *gag* gene. Second, the K222 5′ flanking sequence is only 78% similar to the K111 5′ flanking sequence, known as CER:D22Z3 [10]. The sequence differences in the K222 5′ flanking region and the likely positioning of K222 in the pericentromere domain (see below) led us to designate these repetitive regions pCER:D22Z8. At the 3′ end of K222, however, we identified the target site duplication of K111 (GAATTC) flanked by a CER:D22Z3 element.

After we identified the putative complete genome of K222, we looked for similar sequences in human genome databases. We did not find sequences similar to K222 in the most recent human genome assembly GRCh38/hg38, nor in human Sequence Read Archive libraries. However, we found a K222 provirus in the Whole-Genome Shotgun (WGS) Contigs library (Acc. No. AADC01167561.1). This sequence is from genomic DNA from a presumably healthy person, which suggests that K222 is not only present in the DNA of some cell lines but also in the genomic DNA of healthy modern humans. This K222 sequence was also devoid of the 5′LTR and the *gag* gene, with the deletion occurring at exactly the same position that our PCR and sequencing studies revealed. Interestingly, this K222 sequence is flanked by pCER:D22Z8 elements at both sides and does not have the K111 target site duplication GAATTC, which we identified at the 3′ integration site of K222 from a human cell line. This may indicate that the complete K222 sequence we amplified from a human cell line is a recombinant K222/K111 sequence. The recombinant K222/K111 sequence we amplified is deposited in the NCBI database (Acc. No. KF651980).

As we have found distinct features in the 5′ and the 3′ integration sites of K222 that made it different from K111, we looked for other possible K222 sequences in WGS libraries. We found five more K222 sequences (Acc. Nos. ABSL01025452.1, ABBA01170497.1, AADB02159125.1, ABSL01190241.1, and ABBA01169090). They have a deletion in the LTR and *gag* gene, and therefore show a pCER:D22Z8-*pro* junction. We also identified three more K222 sequences (Acc. Nos. AADB02144450.1.1, ABSL01242357.1, and ABBS01119704.1) that have sequences similar to K222 but not K111 at the 3′ LTR integration site. These sequences were detected in DNA samples from presumably healthy individuals, again suggesting the occurrence of K222 in the human population at large.

**Figure 3:**
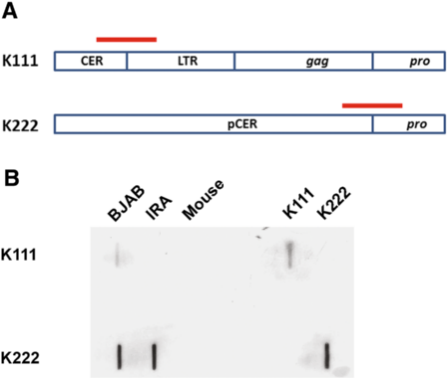
Detection of the K222 provirus in the genome of human cell lines by slot blot analysis. The DNA of human cell lines that were found to have or lack the 5′ end of K111 by PCR, and presumably contain the truncated K222 provirus, were screened for K111 and K222 by slot blot analyses. **(A)** Generation of K111 and K222-specific biotinylated probes. Probes were generated by PCR incorporation of biotin-labeled dCTP. The K111 probe is 422 bp long and spans the CER:D22Z3 flanking sequence and the beginning of the LTR of K111. The K222 probe is 464 bp long and covers the pCER:D22Z8 flanking sequence and *pro* gene of K222. **(B)** DNA from the B-cell lines BJAB (having the 5′ end of K111) and IRA (lacking the 5′ end) as observed by PCR, were screened for K111 and K222 virus by slot blotting. DNA was cross-linked to PVDF membranes and screened for K111 and K222 using biotinylated probes. The probes were detected by chemiluminescence with HRP-conjugated streptavidin. The K111 probe, which targets the 5′ end of genomic K111, reacted with the DNA of BJAB cells but not IRA cells, confirming the lack of the 5′ end of the viral genome in IRA cells. The K222 probe reacted with the DNA of both BJAB and IRA cells, confirming that both cell lines have provirus K222, which is truncated at the 5′ end. Mouse DNA served as a negative control, and plasmids containing either K111 or K222 genomes were used as positive controls. The K111 probe did not react with the K222 plasmid and vice versa.

To confirm the 5′ end deletion of K111 and the existence of K222 observed by PCR, we performed slot and southern blotting in DNA samples of cell lines, one that appeared by PCR to contain K222 but not the K111 provirus, and another cell line that should have both proviruses. We created specific biotinylated probes for K111 and K222 detection as described in Materials and methods. The K111-specific probe is a 422 bp product that spans the 5′ flanking sequence of the K111 provirus and the immediate 116 bp of its 5′ LTR. The K222-specific probe is a 464 bp product that spans the 5′ flanking sequence of K222 provirus and the immediate 396 of its *pro* gene (Figure 3A). In the slot blot, we observed that the K111 probe does not recognize DNA from IRA cells, mouse DNA, or a plasmid containing the K222 sequence (Figure 3B). The K111 probe, however, recognized DNA from BJAB cells. These observations verify our previous findings by PCR (Figure 1A). The K222 probe recognized DNA from both human B-cell lines as well as a plasmid containing a complete K222 sequence, but not a plasmid with K111 or mouse DNA. These observations demonstrate the specificity of the K222 probe and confirm the existence of K222 in human DNA. Using southern blot analyses, we further verified the detection of K222 in the DNA of human B-cell lines that either do or do not have the K111 5′ end (data not shown). The K222 probe recognized DNA from a plasmid containing K222 but not K111, further confirming our observations. These results suggest that K222 indeed is distinct from K111 and may exist in much of the human population.

**Figure 4:**
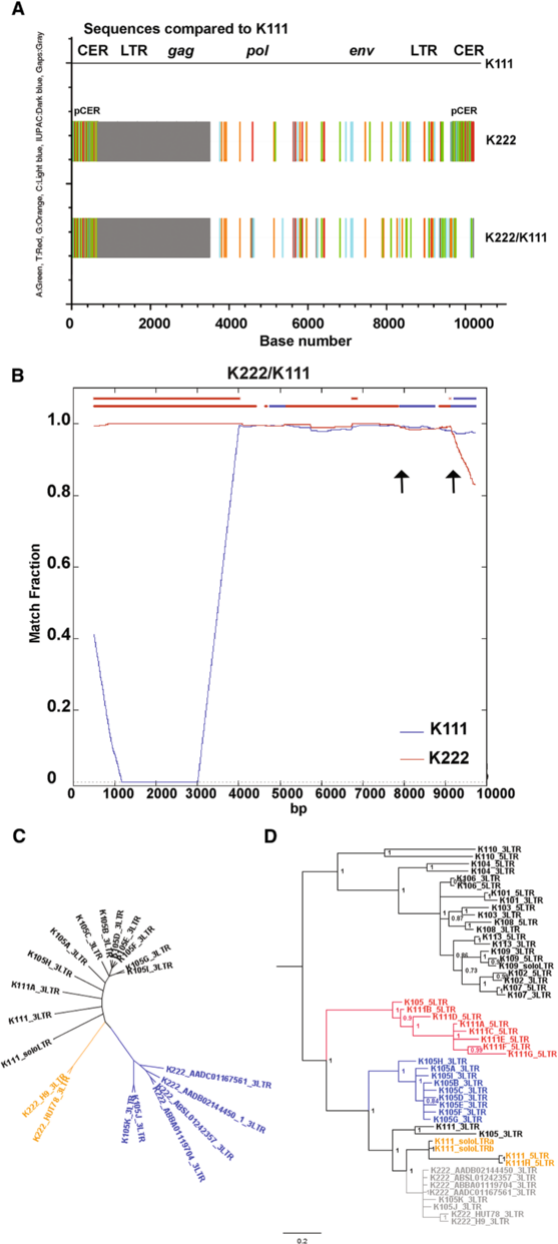
Genomic structure and nucleotide differences of full-length K111, K222, and K222/K111 recombinant proviruses. **(A)** Highlighter plot showing the nucleotide differences between K111 along with K222 provirus found in a WGS database (Acc. No. AADC01167561.1) and K222/K111 recombinant provirus isolated from the genome of the H9 cell line indicated by tick marks (green ticks: A; red ticks: T; orange ticks: G; light blue ticks: C). Gray boxes denote areas deleted in K222. **(B)** Recombination plot of K222/K111 provirus. The similarity between the query K222/K111 recombinant sequence and each parental K222 and K111 provirus is plotted for each position of an approximately 10 Kb bp sliding window. The Y axis represents the match fraction of the query sequence to each parental sequence (red and blue lines). A match fraction of 1 means 100% identity. The recombinant query sequence is illustrated on the X axis (upper red/blue line at the top). Arrows indicate recombination spots. **(C)** A phylogenetic dendrogram displays three major clades; the 3′ LTR K111 (sometimes called K105) sequences previously reported (10; black), 3′ LTR K222 sequences found in human databases (blue), and the 3′ LTR of K222 sequences found in H9 and HUT78 cell lines (yellow). Previous sequences assigned by us as K105J and K105K were indeed K222 sequences and were flanked by pCER:D22Z8 repeat. **(D)** K222 and K111 proviruses arose by independent infections. A Bayesian inference tree shows the clustering of the 5′ and 3′ LTRs from various HERV-K (HML-2) proviruses. The K111 5′ LTR (red) and the 3′ LTRs of K111 (blue) and K222 (gray) proviruses cluster in three independent clades with a common ancestor. Posterior probability values > 70 are shown.

We created an alignment of the full-length K111 and K222 and the recombinant K222/K111 detected in a human cell line. Nucleotide differences between K111 and K222 can be seen using Highlighter plots (Figure 4A). It is noticeable that besides the deletion of the 5′ LTR and *gag* gene in K222, there are differences in the nucleotide sequences flanking either end of both proviruses; K111 flanked by CER:D22Z3 and K222 flanked by pCER:D22Z8. Not apparent in the figure is that the target site duplication GAATTC found at the 5′ and 3′ end of K111, as well as the last 9 bp of the 3′LTR (ACCCCTTCA), are not present in K222. The difference in flanking sequence and premature deletions in K222 suggest that both proviruses arose from two independent infections. As we noted previously, the K222 sequence found in the genome of the H9 cell line has features indicating that it is a K222/K111 recombinant sequence. This K222/K111 sequence has a deletion of the 5′ LTR and the *gag* gene similar to K222 and is flanked at the 5′ end by a pCER:D22Z8 repeat. However, at the 3′ end this sequence has the K111 target site duplication GAATTC and is flanked by a CER:D22Z3 repeat, similar to the K111 provirus (Figure 4A). We performed a recombination test (RIP 3.0) to address whether this K222/K111 sequence arose by recombination. The recombination analysis indicated that this sequence originated for the most part from K222 parental provirus. The 3′ LTR and the flanking sequence next to the integration site, however, clearly resemble the K111 provirus, further suggesting that this sequence is a recombinant K222/K111 provirus (Figure 4B). In addition to this recombination assay, we performed a phylogenetic analysis of several 3′LTRs plus flanking sequence of K111, K222, and recombinant K222/K111 proviruses found in human databases and in our laboratory [10]. The phylogenetic tree shows that K222 LTRs and 5′ flanking sequence found in the CTCL cell lines H9 and HUT78 cluster at a midpoint between K111 and K222 sequences, further indicating that these sequences are recombinant (Figure 4C).

Phylogenetic reconstruction of several HERV-K (HML-2) LTRs, including K111s (sometimes called K105) and K222s, showed that K222 3′ LTRs clustered in the ancestral K111 LTR clade (Figure 4D). At the time of provirus integration, both 5′ and 3′ LTR sequences are similar, but over time they accumulate mutations. The LTRs of individual HERV-K (HML-2) proviruses therefore cluster in a specific clade (for example the LTRs of proviruses K101, K102, and so on cluster together). Likewise, Solo LTRs, which are generated by the recombination of the 5′ and 3′ LTR of full-length proviruses, deleting the internal genes, also cluster to the original 5′ and 3′ LTRs (for example, K109 and K111 Solo LTRs). In this tree (Figure 4D), we observe one clade (shown in black) represented by several distinct HERV-K (HML-2) proviral LTRs indicating independent infections. On the other hand, we observe that K111 LTRs and K222 LTRs have a common ancestral sequence. We further observe that this evolutionary line splits into three well denoted clades: a clade corresponding to the K111 5′ LTRs (red), a clade corresponding to the K111 3′ LTR (blue), and another one derived from K222 3′ LTRs (gray). Of note is that some sequences we amplified in our previous publication [10] and that we labeled K105K and K105J 3′ LTR were actually K222 sequences and do not have the last 9 bp of the 3′ LTR, the GAATTC target site duplication of K111, nor the flanking CER:D22Z3 element. Instead, they are flanked by pCER:D22Z8 element. All these K222 3′ LTR sequences clustered together in an independent branch from the K111 3′ LTR sequences, again suggesting two independent infections. The K222 3′ LTR sequences amplified from H9 and HUT78 lines as well as the K111 solo LTR sequences also cluster close to the K222 3′ LTR sequences but in independent clades (Figure 4D). These lines of evidence suggest that although K111 and K222 appear to represent two different integrations in the germline, at some point during human evolution recombination/gene conversion events between K111 and K222 occurred, which would generate in the phylogenetic tree a common ancestral sequence.

**Figure 5:**
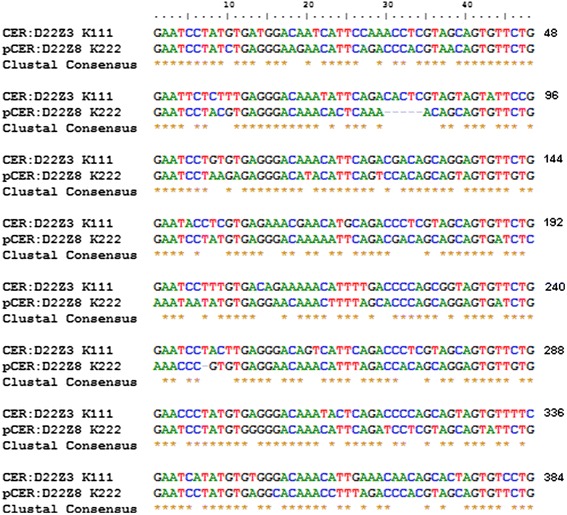
Sequence alignment of CER:D22Z3 and pCER:D22Z8 repeats. Sequences flanking K222 were analyzed. The new sequence repeat we called pCER:D22Z8 shows 71.8% similarity to CER:D22Z3. The repeat pCER:D22Z8 is a centromeric repeat (CER), which we have named pCER according to its likely position in the pericentromere (see the text). The organization of pCER:D22Z8 consists of eight repeats of 48 bp each. According to the chromosomal location of K222, pCER:D22Z8 is located in chromosome 22 and eight additional chromosomes.

Analysis of the integration sequences led us to discover that K111 and K222 inserted into two different centromeric repeats. Both the K111 flanking sequence, the CER:D22Z3, and the K222 flanking sequence, we call pCER:D22Z8, are both the type of CER repeats composed of 384 bp. The organization of these repeats is made of 48 bp segments repeated eight times. The nucleotide similarity of CER:D22Z3 and pCER:D22Z8 is about 71.8% (Figure 5). The similarity between these sequences thus could have allowed us to amplify K222 integration using a set of primers, P1 and P2, which we usually use to amplify the K111 integration.

**Figure 6:**
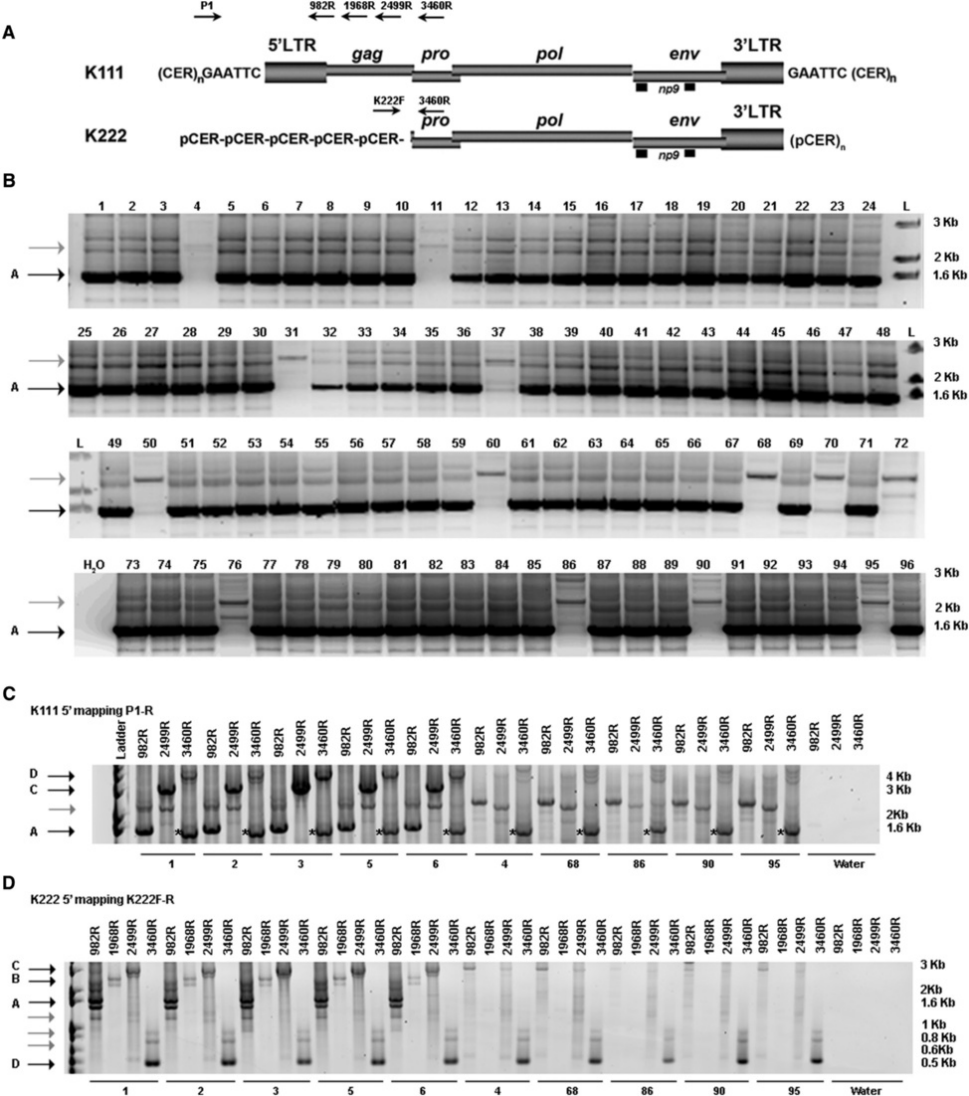
Detection of K111 and K222 in the human population. **(A)** Genomic organization of K111 and K222 proviruses. The location of the primers to map K111 and K222 is shown. **(B)** Detection of K111 5′ end in the human population. The 5′ end of K111 was detected using the primers P1 and P4. The black arrow A indicates the K111 5′ end. The gray arrow indicates non-specific PCR products. On top of each lane is a number signifying each individual subjected to study. **(C, D)** Mapping of K111 **(C)** and K222 **(D)** in five individuals, who are positive or negative for the K111 5′ end, respectively. K111 mapping **(C)** was carried out with primer P1 and reverse primers that bind at positions 982, 2499, and 3460 bp of a K111 provirus. Black arrows indicate specific K111 insertions; A (product P1-982R), C (product P1-2499R), and D (product P1-3460R). The gray arrow indicates non-specific PCR amplifications. K111 detection was observed in the individuals labeled with the numbers, 1, 2, 3, 5, and 6, which are positive for the 5′ K111 end. Non-specific PCR product was detected in individuals labeled with the numbers 4, 68, 86, 90, and 95, which are negative for the 5′ K111 end as shown in B. The primers P1 and 3460R also detect K222 in individuals either negative or positive for the 5′ K111 integration (see stars). K222 mapping was carried out with the primer K222F and reverse primers that bind at positions 982, 1968, 2499, and 3460 bp in reference to K111. PCR products A, B, and C (black arrows) seen in the DNA of K111 positive individuals were shown to be the amplification of K111. No amplification products were seen in individuals lacking the 5′ end of K111. D represents the amplification product of K222.

We found evidence of the occurrence of K222 using the DNA of human cell lines, which lack the K111 5′ integration, and we corroborated the existence of K222 in human WGS sequence databases and by southern blotting. To determine if the absence of the K111 5′ end seen in some human cell lines is also seen in the healthy human population, we attempted to detect K111 using the primers P1 and P4 (Figure 6A). We tested the DNA of 96 human individuals by PCR and found that the K111 5′ integration was not detected in 11 out of 96 individuals (Figure 6B). This suggests the deletion of K111 5′ site is found at a frequency of 11.4% in humans (at least in the population of the United Kingdom studied) and is not a genotype exclusively found in cell lines. In certain individuals that presumably lack the K111 5′ end, we sometimes observe a faint amplification product of the right size. The possibility thus exists that in the genome of these individuals there are a few copies of the K111 5′ end, however the concentration of this product was too low for cloning and/or sequencing confirmation. We further analyzed the K111 5′ deletion genotype with primers that bind along the *gag* gene at positions, 982, 1968, and 2499 in reference to the K101 genome (Acc. No. AF164609.1), and a primer that binds to position 3460 at the *pro* gene, which is present in both K111 and K222 (Figure 6A). We mapped the K111 5′ integration in the DNA of five individuals who tested positive and five more individuals who tested negative for the K111 5′ end. In this K111 5′ mapping, we were able to detect amplification products of K111 in the five patients who tested positive for the K111 5′ end, confirming they have an intact K111 5′ end (Figure 6C). In contrast, we were not able to amplify K111 in five individuals with a negative K111 5′ end (Figure 6C), confirming the absence of the K111 5′ site in this fraction of the human population. Using the primer P1 and the primer 3460R, which binds to the *pro* gene, we detected K111 and K222 in individuals positive for the K111 5′ integration. With this set of primers, we also detected K222 in the DNA of individuals negative for the 5′ integration of K111. Sequence analysis confirmed that in contrast to K111, the K222 sequences amplified have a deletion in the 5′ LTR and *gag* gene.

Finally, we assessed the DNA of individuals having or lacking the 5′ integration of K111 for the presence of K222. We mapped K222 using the primer K222F that binds to the pCER:D22Z8 element and reverse primers targeting either the *gag* gene (missing in K222) or the *pro* gene (present in K222). In the set of individuals with the 5′ K111 end, these sets of primers amplified K111 (Figure 6D). The primer K222F likely sits on the K111 flanking CER:D22Z3 element, which is 71.8% similar to pCER:D22Z8, producing the amplification of K111. These sets of primers, however, did not amplify a provirus in the DNA of individuals with a negative K111 genotype. The combination of primers K222F and the reverse primer that binds to the *pro* gene (3460R) did amplify K222 both in individuals who have K111 and those who do not. Even though the primer K222F could bind to the CER:D22Z3 flanking sequence of K111, in the group of individuals positive for the K111 5′ end these primers instead favor the amplification of K222, and not K111, as targeting K222 will produce the smallest amplification product (Figure 6D). These data thus indicate the existence of K222 in all the humans screened thus far.

### Detection of both K222 and recombinant K222/K111 in individuals missing the K111 5′ integration

**Figure 7:**
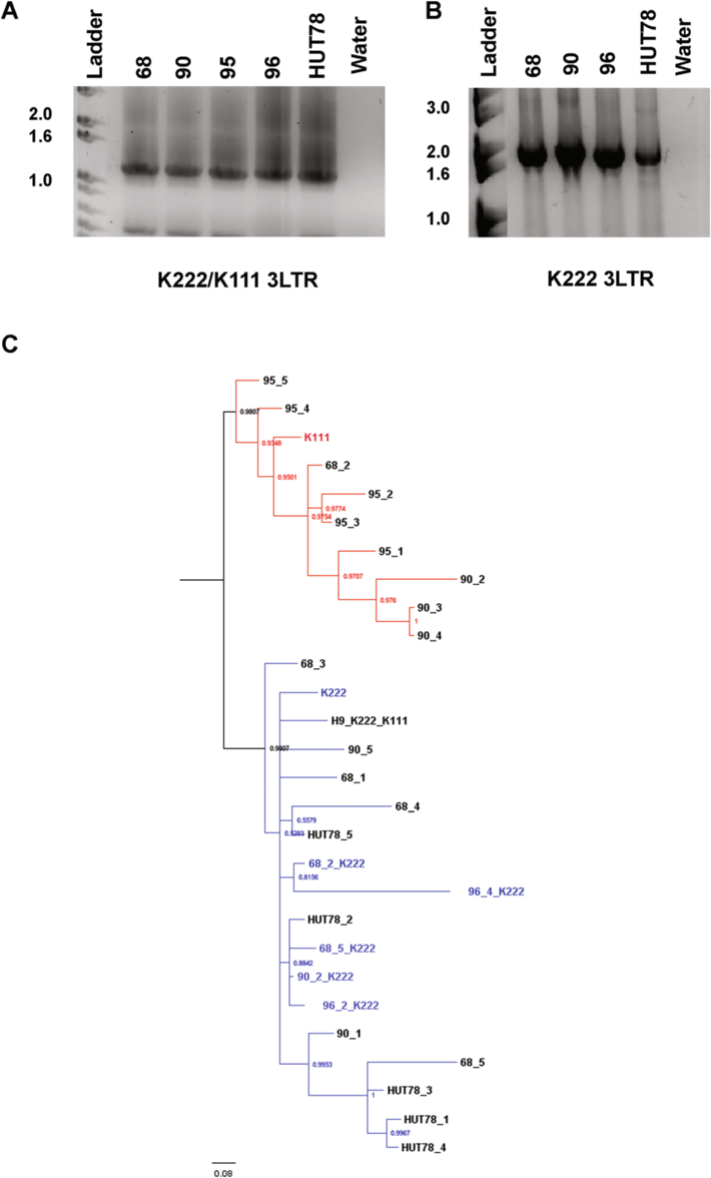
Detection of K222 and recombinant K222/K111 sequences in individuals lacking the K111 5′ end. **(A)** Amplification of K222/K111 recombinant sequences. K222/K111 sequences were amplified with the primer 7972F and the primer P2, which binds to the K111 3′ flanking sequence (see Figure 2) in the DNA from individuals who lack the K111 5′ end (68, 90, and 95) and the cell line HUT78, which also lacks the K111 integration. As a positive control we used the DNA of individual 96, who is positive for K111 5′ end. **(B)** Amplification of K222 3′ integration. K222 was amplified with the primer 7972F and K222LTR-pCER:D22Z8R, the latter primer binding to the LTR-pCER:D22Z8 junction sequence present in K222, but not in K111. K111 3′ integration instead has a 5 bp sequence from the LTR and the target site duplication GAATTC not present in K222. Amplification of K222 3′ integration was seen in individuals having (96) or lacking (68, 90, and HUT78) the K111 5′ end. **(C)** Evolution of K222 and K222/K111 recombinant sequences in humans. A Bayesian inference tree of K222 and K222/K111 LTR sequences obtained by PCR in individuals lacking the K111 5′ end. The K222 sequences amplified are indicated with a K222 label. The tree reveals two different K222 LTR clades; K222 sequences similar to the K222 provirus (blue) and sequences that cluster to the K111 provirus (red). K222 sequences in individuals lacking the K111 5′ end clustering to K111 indicate the likely existence of K111 in the ancestral human lineage of those individuals. The K222/K111 recombinant clade (red) also suggests that K222 and K111 likely recombined by recombination/gene conversion during human evolution before K111 was lost from the lineage. Posterior probability values >85 are shown for the best tree.

The evidence so far suggested that K222 sequences are found in all humans. The K222 sequences we detected in some human cell lines, however, have features of K222 and K111 recombination. We evaluated whether a full K222 sequence (non-recombinant) can be detected in the cell line HUT78 and DNA samples from healthy humans, as well as whether a K222/K111 recombinant sequence can be detected in the human population and not just in cell lines. To accomplish this, we first evaluated possible K222/K111 recombinant sequences using primers that bind to the 3′ integration site of K111 in a set of DNA samples missing or having the K111 5′ end. The primers for this test targeted the *env* gene (7972F) and CER:D22Z3 flanking the 3′ site (P2) (Figure 2A). In samples lacking the K111 5′ end we were able to detect K222/K111 recombinant sequences with these primers (Figure 7A). Confirming previous findings, a K222/K111 recombinant product was also detected in the cell line HUT78. Analysis of these products revealed that these K222/K111 recombinant sequences have the K111 3′ LTR, the K111 target site duplication GAATTC, and the CER:D22Z3 flanking sequence. We next attempted to detect K222 3′ end by designing a primer that binds specifically to the K222 3′LTR-pCER:D22Z8 junction (an area that in the case of K111 would be disrupted by the 3′ LTR end ACCCCTTCA and the K111 target site duplication GAATTC). We were able to detect K222 sequences in individuals positive or negative for the K111 5′ integration as well as in the DNA of the cell line HUT78 (Figure 7B).

If we were not detecting the 5′ end of K111 in some individuals, yet these individuals have recombinant K222/K111 sequences, we asked ourselves whether K111 was present in the ancestral genome before it was deleted. To address this issue, we created a Bayesian phylogenetic tree to determine whether K222 and recombinant K222/K111 sequences split into two different clades. The K222 sequences amplified in individuals with a genotype negative for the K111 5′ end (these sequences are labeled K222) clustered in the clade represented by K222 sequences (shown in blue in Figure 7C). Recombinant K222/K111 LTR sequences in such individuals clustered to either the K111 clade (red) or the K222 clade (blue) (Figure 7C). This suggests that K111 sequences existed in the genome of individuals missing the K111 5′ end. The separation of the K111 and K222 clades also indicates the integration of K222 and the recombination of K222 and K111 occurred at two separate events over the evolution of humans. It is likely that the deletion of K222 at the 5′ end served as a template to delete this area of K111 and produced the K111 5′ end deletion. As many copies of K111 exist in most modern humans, this deletion event mediated by recombination likely happened early in human evolution, before the expansion of K111.

### Integration time of K222 in primate evolution

**Figure 8:**
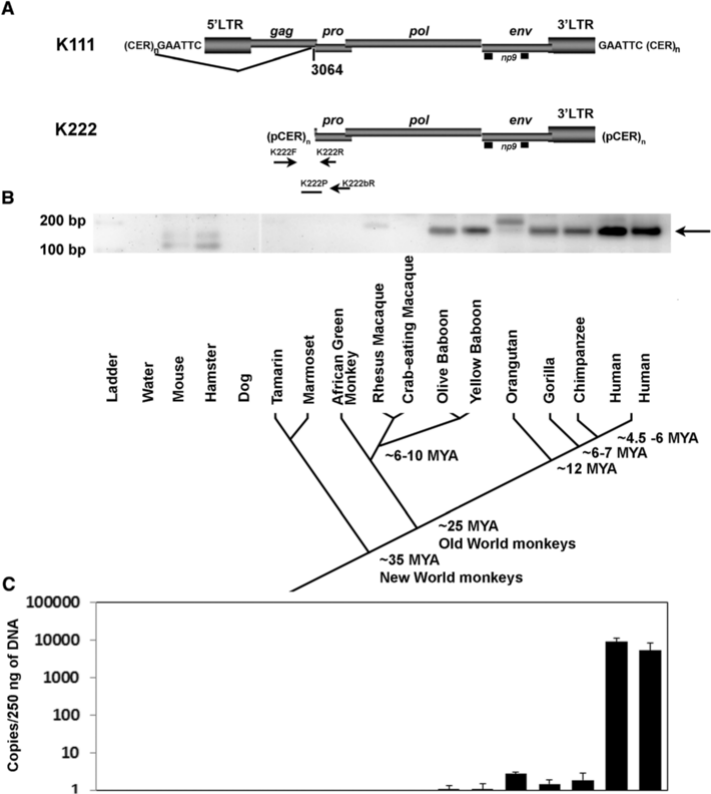
K222 integrated into the primate germline after the divergence of New and Old World monkeys and expanded in copy number during the evolution of humans. **(A)** Genomic organization of centromeric K111 and K222 proviruses. The positions of the primers used to amplify K222 insertions by PCR and qPCR are indicated by arrows. **(B)** Detection of K222 from DNA of New and Old-World primates. K222 was detected by PCR with the primers K222F and K222bR in the baboon, orangutan, gorilla, chimpanzee, and human, but not in macaques, African green monkeys, and New World monkeys. Other bands (for example, the PCR products detected in mouse, hamster, and rhesus macaque) were shown by sequencing to be the result of non-specific PCR amplification. A phylogeny of New World monkeys, Old World monkeys, and hominoids (humans and apes) is shown. Estimated times of divergence are shown. MYA: million years ago. **(C)** Quantitation of K222 copies by qPCR in the genomes of Old World monkeys, humans, and a number of other primates. K222 is likely present as a single copy in the genomes of baboon, orangutan, gorilla and chimpanzee, while present in multiple copies in the human genome. The label of each species in **(B)** matches to the bars.

Despite the sequence similarities between K111 and K222, the differences in the proviral integration sequences, the premature deletions in the genome of K222, and the phylogenetic analysis (see above) suggest that they are distinct proviruses. We attempted to calculate the time of integration of K222 in the germline, and compare this to the time of integration of K111, to elucidate whether these viruses had arisen from independent infections or from a common ancestral infection. Comparison between mutations that differentiate the 5′ and 3′ LTRs has been used to calculate the integration time of a provirus. The sequences of the 5′ and 3′ LTRs are considered identical at the time of integration, but accumulate mutations over time. Thus, by comparing the sequence differences of the 5′ and 3′ LTRs we can estimate the age of viral integration [11]. By comparing the LTRs of K111 we calculated K111 to have entered the germline 2.6 to 6.3 million years ago [10]. However, the 5′ LTR of K222 is missing, and so molecular clock analysis for K222 LTRs is unreliable. Therefore, we searched for K222 integration in the DNA of both New and Old World monkeys and primates using primers specific for K222 (primers K222F and K222bR; Figure 8A), and estimated the integration time of K222 in the primate evolutionary line. We detected K222 in the genome of the baboon (an Old World monkey), orangutan, gorilla, chimpanzee, and human (Figure 8B). Of note, K222 was detected in the genome of all 112 human DNA samples (data not shown). This set of primers did not detect K222 in macaques and African green monkeys (Old World monkeys), New World monkeys, or non-primate species (Figure 8B). We designed other sets of primers that could amplify K222 if existent and again failed to detect K222 in macaques, African green monkeys, and New World monkeys, confirming the previous experiment described above. These data generally suggested that K222 integrated after the divergence of New and Old World monkeys, an event calculated to have happened approximately 25 million years ago (Figure 8B; [29]). While K222 was not detected in macaques and African green monkeys, both Old World monkeys, it was found in the baboon, another Old World monkey that diverged from macaques and African green monkeys about 6 to 10 million years ago (Figure 8B; [30]). These data suggest that K222 was deleted or mutated in the genome of some Old World monkeys. Of course, we might also postulate that K222 was unfixed within the common ancestral Old World monkey population and then fixed only in the baboon but not the macaque and African green monkey genera [31]. In contrast, K111 is only detected in chimpanzee and human DNA [10], suggesting that K111 entered the germline only before the divergence of humans and chimps, an event calculated to have happened approximately 6 million years ago, confirming the molecular clock analysis of K111 LTRs [10]. Therefore, these results indicate that K222 entered the primate line about 25 million years ago and K111 did so about 6 million years ago, indicating that these proviruses arose from independent infections.

**Figure 9:**
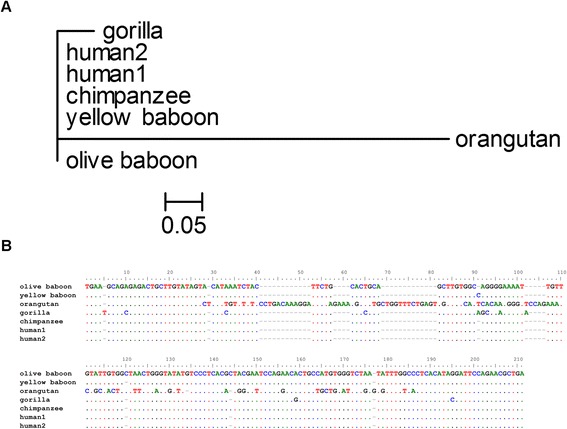
K222 provirus in the genomes of Old World monkeys, primates and humans. **(A)** Phylogenetic neighbor-joining tree of K222 integration sequences amplified from the DNA of baboon, orangutan, gorilla, chimpanzee, and human. The tree is unrooted, with taxa arranged for a balanced shape. The tree was constructed using the Kimura 2-parameter model. The stability of branches was evaluated by bootstrap tests with 10,000 replications. The scale bars represent the nucleotide substitutions per sequence. **(B)** Nucleotide sequence alignment of K222 insertion sequences amplified from the genomes of Old World monkeys, primates, and humans. The sequences are compared to the olive baboon sequence, which is the oldest germline sequence. Dots indicate nucleotide similarities to the master sequence. Nucleotide substitutions are indicated in letters. Several nucleotide insertions can be seen in the sequence of K222 in the orangutan, but not other primates or humans **(B)**, which cause the divergence of the orangutan K222 in the phylogenetic tree **(A)**, suggesting that these insertions arose only during the evolution of modern orangutans.

When amplifying K222 integration, three additional findings were observed. First, K222 amplification in the orangutan produced a PCR product of higher molecular weight than in baboon, gorilla, chimpanzee, and human DNA. Second, sequencing and phylogenetic analysis of K222 amplification products showed that K222 in the gorilla and orangutan diverged from the baboon, chimpanzee, and human cousins (Figure 9A). In the orangutan, K222 incorporated 37 bp nucleotide insertions, which explained the longer PCR product that we observed. K222 sequences accumulated 11 nucleotide substitutions in the gorilla (Figure 9B). Thus, K222 acquired more mutations during the evolution of modern orangutans and gorillas than in the evolution of modern chimpanzees and humans. The third finding, stronger intensity of K222 bands in humans, may indicate expansion of K222 in humans. We performed real-time PCR using equal concentrations of DNA of each species with primers/probe specific for the K222 insertion to estimate an approximate number of K222 copies (Figure 9A). Real-time PCR quantitation estimated that K222 likely exists as a single copy in the genomes of baboon, orangutan, gorilla, and chimpanzee, but multiple copies are found in the human genome (Figure 9C). These data indicate that K222 expanded in copy number during the evolution of humans, sometimes by recombination.

### Determination of K222 copy number in humans

**Figure 10:**
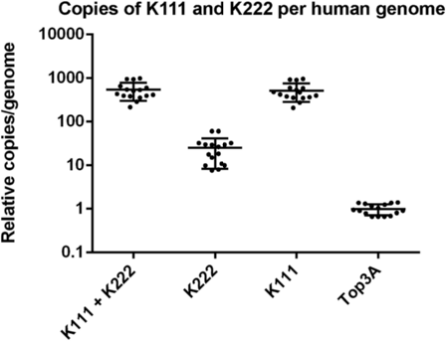
Estimated copy number of K111 and K222 in modern humans. K111 and K222 copy numbers were calculated by qPCR. K111 plus K222 copies were calculated using the primers K111F and K111R and the probe K111P, which detect both K111 and K222. K222 copy number was calculated with the primers K222F and K222R, and the probe K222P, which binds specifically to K222. K111 copies were calculated by subtracting the K222 copies from the K111 + K222 copies. K111 and K222 copies were normalized to the level of the single copy gene TOP3A. The plot indicates the relative copy number per genome of K111 and K222 in 16 healthy individuals.

When we quantitated K222 in the DNA of primates and humans we observed that K222 seemed to exist as a multiple-copy provirus. We next asked what the approximate copy number of K111 and K222 in humans is. We have seen that the quantitation assay we implemented to detect K111 [10] also detects K222. This could be explained by the identical sequence similarity in the *env* region of K111 and K222 we target for that assay (Figure 1A). The assay developed to quantitate K222 is otherwise specific for K222 and does not detect K111 (Figure 8A). We therefore quantitated the K111 + K222 copy number as well as the K222 copy number in 16 individuals using equal amounts of DNA. We further developed a qPCR assay to quantitate the single copy gene Top3A (topoisomerase III A) in the same amounts of DNA as a control. We then normalized the number of copies of K111 and K222 to the number of copies detected for the single-copy gene, TOP3A. The number of copies of K111 was estimated by subtracting the number of copies detected for K222 from the copies detected with the assay that detects both proviruses. Our results indicate that K111 exists in human diploid genomes in on the order of approximately 207 to 968 copies and K222 exists in on the order of eight to 61 copies in the human genome (Figure 10).

### Integration of K222 in human chromosomes

**Figure 11:**
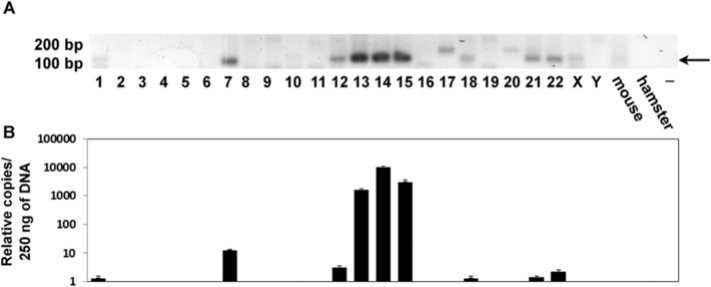
Detection of K222 in human chromosomes. **(A)** K222 was detected by PCR using the set of primers K222F and K222bR in DNA from human/rodent hybrid cell lines, which carry only one specific human chromosome. K222 was found in chromosomes 1, 7, 12, 13, 14, 15, 18, 21, and 22. Other bands (for example the PCR products detected in chromosomes 17, 19, 20, X, and Y) were shown by sequencing to be the result of non-specific PCR amplification. **(B)** Quantitation of K222 copies by qPCR in human chromosomes. The number of K222 copies was calculated from 250 ng of DNA from human/rodent cells lines. Assuming that human cells have between 8 and 61 K222 copies, then we could estimate that about one copy of K222 is present in chromosomes 1, 18, 21, 22, and perhaps more than one in chromosome 12. Several copies of K222, however, exist in chromosomes 7, 13, 14, and 15.

**Figure 12:**
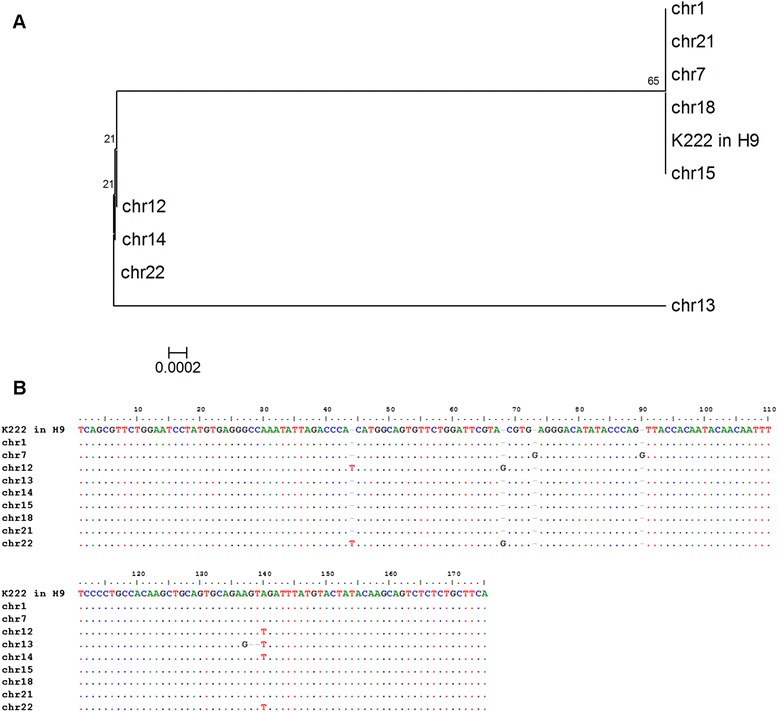
Evolution of K222 in human chromosomes. **(A)** Phylogenetic neighbor-joining tree of K222 integration sequences amplified from the single human chromosomes grown in human/rodent cell hybrids. The tree was constructed in the same way as was the tree in Figure 9. **(B)** Nucleotide sequence alignment of K222 insertion sequences amplified from single human chromosomes. The sequences are compared to the K222 insertion sequence amplified from the genome of the H9 cell line. Dots indicate nucleotide similarities to the H9 sequence. Nucleotide substitutions and insertions are indicated in letters.

K222 exists in the human genome in multiple copies, similar to K111 [10]. In this light, we looked for the presence of K222 in human chromosomes using DNA from human/rodent cell hybrids (Figure 11A), each one harboring one human chromosome. K222 integration was detected by PCR in human chromosomes 1, 7, 12, 13, 14, 15, 18, 21, and 22, but not in any other human chromosome. Interestingly, all centromeres found to harbor K222 also have K111. These DNA were prepared in an outside laboratory, and the possibility of DNA contamination was ruled out by detection of chromosome-specific genes [10]. We next studied whether K222 exists in one or multiple copies in each human chromosome by real-time PCR quantitation. The quantitative assay corroborated the detection of K222 in nine human chromosomes (see above). The quantitative assay also revealed that K222 is likely present in approximately a single copy in chromosome 1, 18, 21, 22, and possibly chromosome 11. K222 exists, however, as several copies in chromosomes 7, 12, 13, 14, and 15 (Figure 11B). Phylogenetic analysis shows K222 sequences clustered in three separate groups: K222 residing in chromosome 13, another K222 group residing in chromosomes 12, 14, and 22, and the K222s found in chromosomes 1, 7, 18, 15, and 21 (Figure 12A). Sequencing of K222 integration (with the primers K222F and K222bR) shows nucleotide differences between K222 proviral sequences found in specific human chromosomes (Figure 12B).

We next investigated whether K222 sequences detected by PCR can be detected by another methodology: deep sequencing and bioinformatics analysis of human DNA samples. We screened for K222 in HERV-K (HML-2)-enriched DNA libraries prepared from splenic fibroblasts and adjacent malignant lymphocytes from a patient with large B-cell lymphoma [10]. We searched for sequence similarities to K222 5′ integration: this is defined as having at least 20 bp of 5′ flanking sequence pCER:D22Z8, the junction sequence ACATATACCCAGT, and 20 bp of the adjacent K222 provirus. We screened for all K222 insertion sequences amplified in nine human chromosomes. Using this independent approach, we detected hundreds of reads of identical K222 integrations in these human DNA libraries (data not shown), confirming the existence of multiple K222 in humans. We detected sequence reads identical to K222 sequences that clustered in three distinct phylogenetic K222 groups, confirming the observations made above using PCR. We further detected several K222 insertion sequences when screening the raw data of sequence read archive (SRA) libraries generated by deep-sequencing studies of human DNA (data not shown). As we noted earlier, we also detected several K222-related integrations in WGS libraries.

### Location of K222 in the chromosome

**Figure 13:**
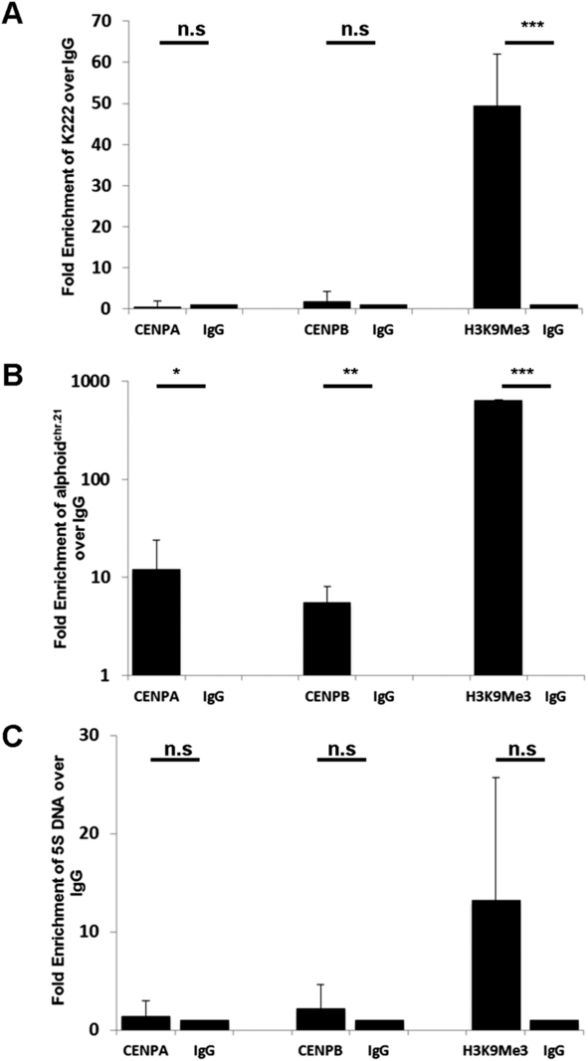
ChIP analysis shows that K222 proviruses are found in pericentromeric regions. Quantitative PCR of K222 DNA, the centromeric 11-mer alphoid repeat of chromosome 21 (alphoidChr.21) DNA, and 5S ribosomal DNA immunoprecipitated by antibodies to CENPA, CENPB, H3K9Me3, or control IgG. **(A)** Compared to the control IgG fraction, K222 is enriched 50-fold in the H3K9Me3 fraction, but not in the centromeric CENPA and CENPB protein fractions. **(B)** The positive control, the alphoid^Chr.21^, is enriched approximately 8-fold in each of the CENPA and CENPB fractions, and approximately 650-fold in the H3K9Me3 fraction. **(C)** The negative control, 5S ribosomal DNA present in the q arm of chromosome 1, shows no significant enrichment with antibodies to CENPA, CENPB, or H3K9Me3. Graphs show the relative enrichment normalized to control IgG-precipitated fractions from three independent experiments. Asterisks indicate statistical significance: *** = *P* <0.001, ** = *P* <0.01, * = *P* <0.05, n.s = not significant.

We next further addressed whether K222 resides in the core or the periphery of the centromere by using chromatin immunoprecipitation (ChIP) studies. The histone 3 variant centromere protein A (CENPA) [32] and the centromere protein B (CENPB) [33] are both binding proteins specific to the centromere core; the histone post-translational modification mark H3K9Me3 is a hallmark of the pericentromeric domain [34]. We immunoprecipitated CENPA, CENPB, and the H3K9Me3 mark in chromatin extracts from HeLa cells using specific antibodies, and K222 integration linked to these centromere marks was then quantitated by qPCR. We did not find enrichment of K222 in CENPA and CENPB immunoprecipitated fractions (Figure 13A), while an enrichment of the positive control for centromeric DNA, the 11-mer alphoid repeat of chromosome 21 (alphoid^Chr.21^), was found as previously reported [10,35]; Figure 13B). As expected, antibodies specific to CENPA and CENPB did not enrich the 5S ribosomal DNA gene (used as a negative control), which is found in the q arm of chromosome 1 (Figure 13C). Immunoprecipitation of the H3K9Me3 histone mark, however, which is found abundantly in pericentromeric regions [34], yielded a marked enrichment of K222 (approximately 50-fold change) and the endogenous alphoid^Chr.21^ repeat (approximately 650-fold change), but did not significantly enrich the 5S ribosomal DNA (Figure 12A to C). These results strongly suggest that K222 sequences reside in the pericentromeric domains of the centromere, and not in the centromeric core. Although we cannot rule out the possibility that K222 exists in other areas of the genome, it appears that at least the vast majority of K222 reside in the pericentromere.

## Discussion

We previously identified a multiple-copy human endogenous retrovirus, K111, which infected the primate germline about 6 million years ago and colonized the centromeres of 15 human chromosomes during the evolution of hominins [10]. In this study, we report the existence of a new multiple-copy endogenous retrovirus we have termed K222. This virus infected the primate germline around 25 million years ago and invaded the pericentromere of nine chromosomes during the evolution of modern humans, but was not previously annotated in the human genome. As in the case of K111, the similarity of K222 proviruses and the repetitiveness of these sequences in several chromosomes may have resembled segmental duplications, which would have rendered them impossible to assemble in the human genome sequence. Despite the similarity of the K222 and K111 proviruses, K222 does not appear to be derived from K111. K222 entered the germline about 25 million years ago, as indicated by the detection of K222 in macaques, baboons, gorillas, and orangutans, species that separated from the primate line before the appearance of K111 6 million years ago. It is likely that soon after K222 integration, the provirus suffered a deletion at the 5′ end, which is evident in the genome of the distant macaques and the modern humans, and explains why no trace of the full element can be found. In addition, although we cannot identify the target site duplication created upon integration of K222, we can recognize the target site duplication of the K111 provirus (GAATTC) at the 5′ and 3′ site, and these sequences are not present in K222, suggesting they are separate infections of the germline. Further, the genome of K222 shows premature mutations such as deletions at the 5′ and 3′ end. Finally, the flanking sequences of K111 and K222 are only 71.8% similar. These findings together with the phylogenetic analysis suggest that K111 and K222 are independent infections of the genome.

K222 is part of the family of human endogenous retrovirus type-K (HERV-K), group (HML-2), which infected the germline on multiple occasions over the last few millions of years of evolution. In contrast to other members of this group of endogenous retroviruses, K222 is represented by insertion in multiple locations throughout the human genome. K222 proviruses can be recognized by two characteristic features in addition to their primary sequence: (1) the 5′ and 3′ flanking sequences of each K222 integration are pCER:D22Z8 repetitive elements; and (2) the provirus genome is devoid of the 5′LTR and the *gag* gene (Figure 6A). Deletion of the 5′LTR, *gag* gene, and the last 9 bp of the 3′LTR of K222, as well as premature stop codon mutations acquired in *pro*, *pol*, and *env* genes, have rendered K222 incompetent to replicate. The expansion of K222 in the centromeres of several human chromosomes during the evolution of modern humans has been achieved then through several recombination events in K222 loci, rather than multiple K222 infections; inactivation of K222 by 5′LTR-*gag* deletion was evident early in evolutionary time (about 25 million years ago).

Expansion of HERV-K (HML-2) loci in the human genome has occurred through two mechanisms. As an example, the tandem repeat HERV-K (HML-2.HOM) or ERV-K6 arose through a process of segmental duplication to exist as two proviral copies sharing one LTR [36]. The expansion of K111 in multiple loci in the human genome may have been produced through a different process: centromere recombination/gene conversion. The latter process seems to have amplified K222 into multiple loci as well. The expansion of K111 and K222 into several human centromeres provides two different lines of evidence of viruses that infected the germline on two separate occasions, separated by 20 million years of evolution, with the integrated proviruses subsequently copied into multiple loci of several chromosomes through centromere recombination processes during the evolution of modern humans. Remarkably, an unrelated lineage of endogenous retroviruses in the kangaroo, (KERV), has also been shown to be copied into multiple loci on several centromeres [37,38].

The sequence of the human centromere is the last major frontier of genomic studies in the Human Genome Project. Highly repetitive elements in the centromere make this area recalcitrant to cloning and sequencing; despite great efforts to overcome these difficulties, the centromere is the largest missing piece of the Human Genome Project, approximately 5% in total [39-41]. Furthermore, centromeric sequences have been difficult to assemble, in part because of the high volume of segmental duplications (sequence fragments longer than 1 Kb in length with more than 90% similarity) obtained by centromere sequencing. DNA segments containing K222 sequence would resemble segmental duplications and consequently would be missed from the current assembly of the human genome [39-41].

In contrast to other areas of the chromosome, recombination between different centromeres was thought to be inefficient [42,43]. Nonetheless, evidence for recombination, at least between sister centromeres, has been found [44]. The distribution of large arrays of repetitive elements in the centromere suggests that recombination through gene conversion is a common occurrence within centromeres [45]. It is thus conceivable that repetitive elements undergo extensive growth and regression because of unequal recombination events [46]. Copying K111 and K222 in several centromeres suggests that recombination/gene conversion events took place within and among the centromeres during human evolution. The sites of the actual recombination events that resulted in K111 and K222 expansion are unclear at this time. However, we have shown strong evidence suggesting recombination events between K111 and K222 during evolution. Sequence evidence suggests that areas of the provirus genome, as well as the flanking repeats, would have participated in this process: (1) Some of the K222 3′LTR sequences show evidence of recombination with K111 LTR, even in people with a genotype negative for K111 5′ LTR; (2) we have found recombinant K222/K111 sequences that contain the 3′ LTR of K222 but are flanked by the target site duplication and flanking sequences of K111, the GAATTC, and the CER:D22Z3; (3) the sequence of K111 and K222 genes are both approximately 99% similar to each other, but only 95% similar to other HERV-K (HML-2) proviruses, suggesting events of gene conversion between both proviruses; (4) all K222 loci exist in the centromere of chromosomes, the same location where most K111 proviruses are found. These clues indicate that K222 expansion in the pericentromeric domains of the human genome involved recombination among and between K222 proviruses, centromeric K111 proviruses, and their flanking sequences.

The location of K222 proviruses in the centromere, particularly in the pericentromere domain of the human genome, is based on four observations: (1) *in situ* hybridization analysis of centromeric repeats known as CER elements; including the pCER:D22Z8 and CER:D22Z3 have demonstrated that these elements are found in the centromeres of chromosomes 21 and 22 [47,48]. We have named the flanking sequence of K222 pCER:D22Z8, based on the nomenclature for CER repeats in chromosome 22; (2) BLAST analysis of pCER:D22Z8 elements, which flank the 5′ and 3′ sequence of K222, reveals sequences >95% similar to repetitive elements found in several human centromeres; (3) centromeric regions make up part of the 5% of the human genome that has not yet been assembled in the human genome draft sequence [39,41], making this a logical place to look for and find previously unreported repetitive elements; (4) the existence of K222 sequences in centromeres, specifically in the pericentromere domain, has been confirmed using chromatin immunoprecipitation assays (Figure 13). While K111 exists in all the nine chromosomes in which K222 is integrated, in addition to other chromosomes, K111 appears to be in both the pericentromeric and centromeric core domains, whereas K222 appears to be found integrated solely in pericentromeres. Sequence differences found in K222 proviruses will serve to better annotate the pericentromere of these chromosomes, whereas K111 sequences may serve to better characterize the sequences of centromeric cores. Taken together, the sequences of K111 and K222 might well prove useful in studying the centromeric core and pericentromeric domains of the centromeres of each human genome and their interactions. Indeed, one of the most striking findings of the work presented here is the observation that based on analysis of the flanking sequences of K222/K111 recombinant provirus, crossing-over events, and not just gene conversion, took place between and/or within the centromeres of different chromosomes. This observation provides some of the first evidence to suggest that true recombination shapes the centromeres of human chromosomes.

## Conclusions

By studying the genome of certain human cell lines, which lack K111 5′ end, we have discovered a lineage of human endogenous retroviruses with the potential to guide future studies of the DNA sequence and function of pericentromeres. Using human/rodent chromosomal hybrids, we have located K222 proviruses in the pericentromere of nine human chromosomes. Further studies are under way to amplify full-length K222 sequences in each human pericentromere. While additional studies are needed, informative nucleotide substitutions of the K222 provirus present in each pericentromere could provide new geographic points to help annotate pericentromeric sequences and understand the biology of these areas of the human genome. Along with K111, these K222 proviruses are islands of endogenous retroviruses in a sea of repetitive elements that may become reference points to better order the human centromere.

In sum, we have identified two ancestral retroviruses, K111 and K222 that infected the primate germline and became positioned in the modern human centromere. These viral sequences subsequently expanded into multiple copies during the evolution of modern humans. Not only were these sequences copied many times within each centromere, but more surprisingly into the centromere of multiple other chromosomes, events likely mediated by homologous recombination and gene conversion. Copying of K111 and K222 sequences therefore illuminates the vast amount of exchange of genetic material between human centromeres during evolution, regions of the chromosome previously thought to be refractory to recombination. The biology and function of centromeric cores and pericentromeric domains, which are crucial to the proper partitioning of chromosomes, is vital to understanding genetics and cellular function in health and disease.

## Materials and methods

### DNA samples

DNA was extracted using the DNeasy blood and tissue kit (Qiagen) from B-cell lines (BJAB, IRA, and SA5), CTCL cell lines (H9, HUT78, and H9/HTLVIII), T-cell Leukemia cell lines (ACH, OM10-1, 8E5, MT2, MT4, SupT1, CEM, HOS-CD4, A3.01, and Jurkat), and teratocarcinoma cell lines (NCCIT and Tera-1). The karyotype of the H9 cell line derivative of HUT78 according to the American Type Culture Collection (ATCC) is as follows: A near triploid cell line (modal number = 69; range = 58 to 74). The frequency of higher ploidies is 2.5%. The line has an extremely complex karyotype with nearly 60% of the chromosomes in each cell being structurally altered marker chromosomes. Among the markers are t(3p4q), t(5q6q), t(5p6p), i(18q), i(18p); t(4q7p), and del(7)(q32). The first four of these are usually paired. Normal N4, N5, N6, N7, N10, N13, N18, N19, N20 an X are absent.

DNA samples from people of diverse origin were obtained from the blood of 96 Caucasian people (HRC2 Human Random Control DNA panel 2, Sigma Aldrich) and from DNA extracted using the DNeasy blood and tissue kit (Qiagen) from the peripheral blood lymphocytes of 17 HIV-1 patients and 67 cancer patients recruited at the University of Michigan and the North Shore University Hospital following protocols approved by the respective Institutional Review Boards and carried out in accordance with the Helsinki declaration.

DNA from New World and Old World monkeys and primates was obtained from Texas Biomedical Research (San Antonio, TX, USA) and met all the requirements of CITES (Convention on International Trade in Endangered Species of Wild Fauna and Flora) and the Department of the Interior, U.S. Fish and Wildlife Service.

DNA samples from single human chromosomes grown in Human/Rodent somatic hybrid cell lines and the parental rodent cells were obtained from a human chromosomal DNA mapping panel (NIGMS Human/Rodent Somatic Cell Hybrid Mini Mapping Panel # 2 DNA Coriell Cell Repositories).

### Mapping full-length K222

A full-length proviral genome of K222 was amplified from human H9 and HUT78 cell lines and some human DNA samples, in which the centromeric K111 5′ end was not detected using the Expand Long Range dNTPack PCR kit (Roche Applied Science). PCR reactions contained 50 ng genomic DNA, 2.5 mM MgCl_2_, 500 μM dNTPs, 300 nM of each primer, and 3.5 units of Expand Long Range Enzyme Mix. The full-length form of K222 was amplified using either the P1 forward primer or the P2 reverse primers, which bind centromeric repeats, and primers that overlap the HERV-K (HML-2) *gag*, *pro*, *pol*, and *env* genes as depicted in Figure 2 (see Table 1). The position of each HERV-K (HML-2) primer is indicated by the base number where they would bind to the reference K111 genome (Acc. No. GU476554.2). Amplification of K222 3′ integration was performed using the primers 7972F and K222LTR-pCER:D22Z8R; the last primer binds uniquely to K222 and not to the K111 3′ integration. This primer spans the pCER:D22Z8 integration sequence and the 3′LTR of K222. In this region K111 has a 15 bp insertion corresponding to the end of the 3′ LTR (ACCCCTTCA) missing in K222, and the specific K111 target site duplication GAATTC. Amplification of recombinant K222/K111 3′LTR sequences was performed with the primers 7973F and P2, specifically accomplished in patients who lack the K111 5′ insertion.

**Table 1:** **List of primers**

P1	5′-ACA TTC AGA CCA TGG TAG CCG TGT −3′
P2	5′-ACA GTG CTG TGT GGG TCT GAA TGA −3′
P4	5′-GTA CCT TCA CCC TAG AGA AAA GCC T −3′
P9 (283–306)	5′-CAG GAG TGT TCT GGA ATC CTA TG-3′
KLTR 116-94R	5′-AAC AGA ATC TCA AGG CAG AAG AA-3′
K111F	5′-AAG AGC ACC AGG ATG CTT AAT GCC-3′
K111R	5′-AGT GAC ATC CCG CTT ACC ATG TGA-3′
K111P	5′-FAM-TGC CGG TCC TAA CAG TAG ACT CAC-BHQ1-3′
K222F	5′- CAG CGT TCT GGA ATC CTA TGT-3′
K222R	5′- TGT ATT GTG GTA ACT GGG TAT ATG T-3′
K222P	5′-FAM- ACC CAC ATG GCA GTG TTC TGG ATT-BHQ1-3′
K222bR	5′-GAA GCA GAG AGA CTG CTT GTA TAG-3′
K222LTR-pCER-D22Z8R	5′-TCC CTC ACA TAG GGT TGC CCC T-3′
5SDNA-F1	5′- CCG GAC CCC AAA GGC GCA CGC TGG-3′
5SDNA-R1	5′- TGG CTG GCG TCT GTG GCA CCC GCT-3′
11-10F	5′- AGT TTT TAT GGG AAG ACA TTC CCT-3′
Mcbox-3R	5′- CGG GAA TAT CAT CAT CTA AAA TCT-3′
GAPDHF	5′-TGC ACC ACC AAC TGC TTA GCA CCC-3′
GAPDHR	5′-CTT GAT GAC ATC ATA TTT GGC AGG-3′
TOP3AF	5′-ACT AGG TCA GAG ACC CTT ACT G-3′
TOP3AR	5′-CAA GGA GAG GCA GTG ACA AA-3′
982R	5′-GTA CCT TCA CCC TAG AGA AAA GCC T −3′
1968R	5′-TCC AGG TGG CAT CGG TTC TAA CAT-3′
2499R	5′-TTG AGC AAC ATC TTG GAG CCT TGC-3′
3460R	5′-ACT TGC CCA ATA TGC AGC CTT TCC-3′
3500R	5′-TAA TGG CCT TAC ACA CAG GTT TG-3′
4734R	5′-TGC CTA GAG TTG GCC GAA TCC AAT-3′
5388R	5′- GAC TGC AAC CAA CTC TGC TCT TTG-3′
6609R	5′-ATC CCT TCT TCC TCA GGT TTG GCA-3′
7320R	5′-TCC TAA TGT GGT ATG AGG CTG CAG T-3′
1965F	5′-ATG GAA CCG ATG CCA CCT GGA-3′
2641F	5′-AAA GCC TGT GAT GGA ATC GGA GGA-3′
3170F	5′-TTC CCC TGC CAC AAG CTG C-3′
4080F	5′-TCT CGC CTT GGA ATT CTC CTG TGT-3′
5359F	5′-TCG GCT CAA AGA GCA GAG ATG GTT-3′
6586F	5′-TGC CAA ACC TGA GGA AGA AGG GAT-3′
7972F	5′-AAA TTT GGT GCC AGG AAC TGA GGC-3′

PCR was performed using an initial step of 94°C for 2 min followed by 40 cycles consisting of denaturation at 94°C for 30 s, annealing at 55°C for 30 s, and extension at 68°C for 10 min. PCR products were electrophoresed in 0.8% Agarose gels, extracted, and purified with the Zymoclean™ Gel DNA Recovery Kit (Zymo Research), and directly sequenced. Full-length K222 sequences were assembled using CAP (Contig Assembly Program) in the BioEdit platform. *In silico* distribution of pCER:D22Z8 elements flanking the K222 insertion was screened using Repeat Masking from the Genetic Information Research Institute. K222-related proviral insertions were detected in the DNA of all 17 HIV-1 patients, 67 cancer patients, and 96 Caucasian control subjects. Integrity of the DNA samples was measured by positive amplification of GAPDH and K222. A sequence of provirus K222 is found in the NCBI database (Acc. No. AADC01167561.1). A consensus sequence of recombinant K222/K111 is deposited in the NCBI database with Accession Number (KF651980).

### Detection of K222 in DNA samples

Specific amplification of K222 was performed by PCR using the primer K222F, which binds specifically the 5′ flanking pCER:D22Z8 repeat of K222, and the reverse primer K222bR, which binds the HERV-K *pro* gene to produce an amplification product of approximately 171 bp (see Table 1). The location of the primers can be seen in Figure 6A (see Table 1). The PCR was carried out in 45 cycles consisting of 15 s of denaturation at 95°C and 1 min of annealing/hybridization at 60°C, and the products were run out on a 2.5% agarose gel by electrophoresis. K222 PCR product varies in size in the genome of the modern Orangutan because of 37 bp nucleotide insertions (Figure 8B). Specificity of the PCR products was confirmed by sequencing.

### Real-time qPCR specific for K222

The copy number of K222 in cellular DNA was measured by qPCR using a probe that specifically discriminates the K222 pCER:D22Z8-*pro* boundary (Figure 8A). The qPCR was performed as described [10], using the primers K222F and K222R and the FAM-labeled probe K222P. The PCR was carried out in 45 cycles consisting of 15 s of denaturation at 95°C and 1 min of annealing/hybridization at 60°C. The K222 copy number was estimated using serial dilutions of purified K222 PCR product. This K222 PCR product was amplified and sequenced from the DNA of the H9 cell line with the forward primer P1 and a reverse primer that binds the HERV-K (HML-2) *pro* gene at base 3500 in reference to the K111 genome (Acc. No. GU76554.2). The DNA copy number of the PCR product was estimated by reading the DNA concentration at a wavelength of 260 nm using UV spectrophotometry. The purity of the PCR product was estimated by reading the absorbance at wavelength 260 nm and 280 nm in the UV spectrophotometer and estimating the 260/280 ratio, which was approximately 1.8. The specificity of the probe was assessed using DNA samples containing K222 provirus in their genome (human, primates, and Old World monkey) or that lack K222 (New World monkeys, rodents). The relative copy number of K222 per genome in human DNA was estimated in reference to the quantitation of the gene topoisomerase III A (TOP3A), which exists as a single copy in the human genome [49]. Quantitation of TOP3A was performed using the primers TOP3AF and TOP3AR. The TOP3A copy number was calculated using serial dilutions of purified TOP3A PCR product as described above. The relative copy number of K222 was calculated by dividing the number of copies of K222 from the number of copies detected of TOP3A in equal amounts of cellular DNA.

### Chromatin immunoprecipitation (ChIP)

ChIP assays to assess the association of centromeric proteins and heterochromatic marks with K222 were performed using the ChIP-IT™ Express Enzymatic kit (Active Motif) following the procedures described by the manufacturer. Briefly, approximately 70% to 80% confluent HeLa cells grown in 15 cm plates were fixed with 1% paraformaldehyde to cross-link protein to DNA. HeLa cells were lysed and the chromatin was sheared with an enzymatic solution. Chromatin was immunoprecipitated overnight using specific monoclonal antibodies (Abcam) to CENPA (ab13939), CENPB (ab134144), and the heterochromatic histone mark H3K9Me3 (ab10812), or with non-specific IgG antibodies. Centromere and heterochromatin occupancy on target K222, meaning the K222 sequences bound to the centromeric proteins or heterochromatin histone marks (CENPA, CENPB, and H3K9Me3), was measured by qPCR. The fold enrichment was determined based on the cycle differences (ΔCt) between the sample vs. control (IgG). The 11-mer alphoid repeat of chromosome 21 (alphoid^chr.21^) served as a positive control for centromeric sequences, and was amplified with the primers 10-10F and mcbox3R [34]. 5S ribosomal DNA served as a negative control in the centromere studies, as this gene localizes to the q arm of chromosome 1. Primer sequences used for ChIP are listed below.

### *In silico* sequence analysis

The K222 sequences amplified with primers K222F and K222bR in the DNA of human and other animal genomes, and the DNA from human/rodent chromosomal cell hybrids were BLASTed to the NCBI database. The sequences were aligned in BioEdit and exported to the MEGA 5 matrix. Phylogenetic trees were constructed by the neighbor-joining method, using the statistical bootstrap test (10,000 replicates) of inferred phylogeny and the Kimura-2 parameter model [50,51]. LTR trees were generated using Bayesian inference (MrBayes v. 3.2; [52,53] with four independent chains run for at least 1,000,000 generations until sufficient trees were sampled to generate more than 99% credibility. Highlighter plots were generated using the highlighter tool of the Los Alamos HIV sequence database. The potential recombinant sequences were verified using phylogenetic analysis of Bayesian inference and the parent sequences identified using RIP 3.0. This program used a sliding window (200 bp in this study) that moves over an alignment containing the query sequence and all of the possible parental proviruses. Best matches are marked if they are significant by using an internal statistical test. We verified the sequence similarity between the putative parent and query sequences on each side of the recombination spot. On several occasions, recombinant sequences were more than 99% similar to each parental sequence.

### Deep-sequencing

Healthy fibroblast and lymphoma B-cells were grown from the splenic tissue harvested from a patient with diffuse large B-cell lymphoma as described [10]. The procurement of this splenic tissue was per protocol approved by the Institutional Review Board of the University of Michigan. Genomic DNA was extracted from the above cells using the Qiagen Blood and Cell Culture DNA Midi Kit (Qiagen), with purity confirmed by spectrophotometry. Paired-end libraries were prepared from the genomic DNA samples, and HERV-K (HML-2) was enriched by hybridization with a probe set spanning a consensus sequence of the full-length HERV-K (HML-2) LTR. The hybridized DNA was then captured, washed, and re-amplified prior to deep sequencing using the Illumina HiSeq 2000 system. The deep sequencing data were deposited in the SRA database with Accession codes SRX958815 and SRX959907.

### Bioinformatics analyses

K222 insertions were analyzed in HERV-K (HML-2) LTR-enriched libraries created in our laboratory, from sequence read archive (SRA) stores obtained by next-generation deep-sequencing projects of human and other primate genomes. Sequence reads were aligned to the human reference genome and to K222-related sequences obtained by PCR (see above) using BOWTIE, allowing no more than 1 bp mismatch. To detect K222 sequences, we screened the libraries for sequence reads that contained at least 20 bp of the pCER flanking sequence, the recombination spot sequence ACATATACCCAGT, and at least 20 bp of the adjacent K222 provirus, allowing no more than 1 bp mismatch. Sequence reads that matched these three criteria were retained for visual examination. In addition, we screened for read sequences that uniquely hit each of the K222-related sequences in each human chromosome but did not hit the human reference genome. A relatively conservative criterion of > =3 unique hit locations on target in both control and tumor samples was used to ensure that the identified targets were not due to random sequencing errors.

### Generation of K111 and K222 biotinylated probes

K111 and K222-specific biotinylated probes were generated by PCR using the FastStart Taq DNA polymerase dNTP Pack (Roche) as described by the manufacturer, and incorporated biotin-14-dCTP (Life Sciences). The reaction contained 50 ng of human DNA, 0.2 μM of each specific primer, 2 mM of MgCl_2_, 40 μM of each dNTP (dATP, dTTP, dGTP), 10 μM of unlabeled dCTP and 30 μM of biotin-14-dCTP, and 2 U FastStart Taq DNA Polymerase. K111-specific probe was generated with the primers P9 (283–306) and KLTR 116-94R, which amplify a 422 bp product that spans the 5′ flanking sequence of K111 provirus and the immediate 116 bp of its 5′ LTR (see Table 1). The K222-specific probe was generated using the primers K222F and 3460R, which amplify a 464 bp product that spans the 5′ flanking sequence of K222 provirus and 396 of its *pro* gene (see Table 1). PCR products were purified using the DNA Clean and Concentrator Kit (Zymo Research).

### Southern/Slot blotting

Five micrograms of human DNA were digested with H*ind*III overnight. DNA was electrophoresed in a 0.8% agarose gel for 18 h at 30 volts. The gel was denatured in 1.5 M NaCl, 0.5 M NaOH. The DNA was transferred onto a nylon/PVDF membrane using upright capillary transfer in 20X Sodium Chloride/Sodium Citrate (SSC) blotting buffer for 18 h. For the slot blot, 5 μg of human DNA was directly applied onto a PVDF membrane using a slot blot manifold (Hoefer Scientific Instruments) and applying vacuum. Blots were washed in 2X SSC, dried at room temperature, and the DNA fixed for 2 min under UV light. Blots were incubated in prehybridization/hybridization solution (6X SSC, 5X Denhardt’s solution, 50% Formamide, 0.5% Sodium Dodecyl Sulfate (SDS), and 10 mg/mL sonicated-salmon sperm DNA) for 1 h. Blots were then incubated with approximately 100 ng/mL of denatured biotinylated probe in hybridization solution at 42°C overnight. Blots were washed twice in 2X SSC, 0.1% SDS for 10 min at room temperature, and two more times in 0.1 X SSC, 0.1% SDS for 10 min at 65°C. Blots were incubated in blocking solution (phosphate buffered saline (PBS) with 5% Bovine Serum albumin (BSA) and 0.1% Tween) for 1 h. Biotinylated DNA was detected with Thermo Scientific Pierce NeutrAvidine-HRP (Pierce, 1:10,000 dilution) in blocking solution for 1 h. Blots were washed three times in PBS, 0.1% Tween. Chemiluminescence detection was achieved with the SuperSignal West Pico Chemiluminescent Substrate Pierce and autoradiography with X-ray films. Ten nanograms of plasmids containing either K111 or K222 genomes served as positive controls.

### Statistical analysis

The relative enrichment of K222 DNA associated with centromere (CENPA and CENPB) and pericentromere proteins/marks (H3K9Me3) in ChIP experiments (using IgG as a control antibody) was compared using the student’s *T* test. Two-tailed *P* values were considered significant at *P* <0.05.

## Abbreviations

CENPA: Centromere protein A
CENPB: Centromere protein B
CER: Centromeric repeat
ChIP: Chromatin immunoprecipitation
CTCL: Cutaneous T-cell lymphoma
DNA: Deoxyribonucleic acid
H3K9Me3: Histone 3 trimethyl lysine 9
HERV-K: Human endogenous retrovirus type K
HIV-1: Human immunodeficiency virus type-1
KERV: Kangaroo endogenous retrovirus
LTR: Long terminal repeat
NCBI: National Center for Biotechnology Information
pCER: Pericentromeric repeat
PCR: Polymerase chain reaction
SRA: Sequence read archive
TOP3A: Topoisomerase 3A
UV: Ultraviolet
VLPs: Virus-like particles
RNA: Ribonucleic acid
